# Partial pulpotomy without age restriction: a retrospective assessment of permanent teeth with carious pulp exposure

**DOI:** 10.1007/s00784-021-04007-2

**Published:** 2021-06-02

**Authors:** Florin Eggmann, Thomas J. W. Gasser, Hanjo Hecker, Mauro Amato, Roland Weiger, Lucia K. Zaugg

**Affiliations:** 1 Department for Periodontology, Endodontology and Cariology, University Center for Dental Medicine UZB, University of Basel, Mattenstrasse 40, CH-4058 Basel, Switzerland; 2 Clinic of Reconstructive Dentistry, Center of Dental Medicine, University of Zurich, Zurich, Switzerland; 3 Private Dental Practice, Basel, Switzerland; 4 Department of Reconstructive Dentistry, University Center for Dental Medicine Basel UZB, University of Basel, Basel, Switzerland

**Keywords:** Vital pulp therapy, Dental caries, Pulpotomy, Pulpitis, Mineral trioxide aggregate

## Abstract

**Objectives:**

This study aimed to retrospectively evaluate clinical and radiographic outcomes of partial pulpotomy performed in permanent teeth with carious pulp exposure.

**Materials and methods:**

Records of patients undergoing treatment at an undergraduate dental clinic between 2010 and 2019 were screened for partial pulpotomies in teeth with a presumptive diagnosis of normal pulp or reversible pulpitis. The follow-up had to be ≥ 1 year. Patient data were retrieved and analyzed using Mantel-Cox chi square tests and Kaplan–Meier statistics. The level of significance was set at α = 0.05.

**Results:**

Partial pulpotomy was performed in 111 cases, of which 64 (58%) fulfilled the eligibility criteria. At the time of partial pulpotomy, the mean age was 37.3 (± 13.5) years (age range 18–85). The mean observation period was 3.1 (± 2.0) years. Two early failures (3.1%) and five late failures (7.7%) were recorded. The overall success rate of maintaining pulp vitality was 89.1%, with 98.4% tooth survival. The cumulative pulp survival rates of partial pulpotomy in patients aged < 30 years, between 30 and 40 years, and > 40 years were 100%, 75.5%, and 90.5%, respectively, with no significant difference between the age groups (p = 0.225). At follow-up, narrowing of the pulp canal space and tooth discoloration were observed in 10.9% and 3.1% of cases, respectively.

**Conclusions:**

Across age groups, partial pulpotomy achieved favorable short and medium-term outcomes in teeth with carious pulp exposure.

**Clinical relevance:**

Adequate case selection provided, partial pulpotomy is a viable operative approach to treat permanent teeth with deep carious lesions irrespective of patients’ age.

## Introduction

Different operative treatment approaches are available to manage deep carious lesions, which are defined by a radiographic involvement of ≥ 75% of the total dentin thickness [1]. In the absence of signs and symptoms of irreversible pulpitis, indicative of either partial or total necrosis of the coronal pulp, a primary goal of these treatment approaches is to maintain the vitality and health of pulp tissue [2, 3]. The advantages of preserving pulp vitality are legion: the dentin-pulp complex may continue to fulfill its developmental, defensive, and proprioceptive functions, the long-term tooth prognosis is more favorable, and—adequate case selection provided—it can improve the cost-effectiveness of the treatment [2, 4, 5].

Stepwise and selective caries removal aim to avoid pulp exposure and to alter the microbial ecology of the caries biofilm, with current evidence suggesting one-stage selective caries removal to be more advantageous compared with stepwise caries removal in most cases [6]. However, pulp exposure is inevitable in some cases, in particular when nonselective carious tissue removal to firm dentin is performed [7]. In such cases, vital pulp therapy treatment modalities may offer a viable alternative to root canal treatment [8]. Vital pulp therapy includes direct pulp capping, partial pulpotomy, and complete pulpotomy [9].

Histologic studies demonstrated that, in teeth with cariously exposed vital pulps, irreversible pulp tissue damage is frequently confined to the area of the pulp beneath the carious lesion [3, 10]. A partial pulpotomy consists of the amputation of 2 to 3 mm of coronal pulp tissue subjacent to the site of pulp exposure and the subsequent placement of a biocompatible or bioactive capping material [7]. In contrast to direct pulp capping, partial pulpotomy removes pulp tissue that may be infected or display necrosis or microabscesses. Consequently, the chance of pulpal healing is higher [7, 9]. Likewise, complete pulpotomy may be carried out to remove irreversibly damaged coronal pulp tissue [9]. Partial pulpotomy is, however, considered the more conservative approach [7]. Unlike complete pulpotomy, it preserves some of the cell-rich tissue of the coronal pulp, thus enabling the ongoing deposition of cervical dentin and reducing the likelihood of root canal obliteration [7].

A recent meta-analysis reported high success rates of partial pulpotomy in posterior permanent teeth with carious pulp exposure: the random-effects model, analyzing data from the seven clinical trials meeting the inclusion criteria, gave a success rate of 92% at the 2-year follow-up [7]. The preoperative pulp status was the only variable significantly associated with the success rate at the 1-year follow-up [7]. The outcome in teeth with a presumptive clinical diagnosis of irreversible pulpitis was significantly inferior compared with teeth that were given a presumptive diagnosis of normal pulp or reversible pulpitis [7].

The pulp tissue of young patients is more cellular and has been considered to have a higher healing capacity compared with older patients [9]. Yet, according to meta-analytic data, neither the patient’s age nor the stage of the root development impacts the outcome of the partial pulpotomy [7]. It is, however, important to take into account that past clinical studies investigated partial pulpotomy frequently in study populations with a low average age. For instance, the mean age in the studies analyzed in a recent systematic review ranged from 9 to 30.3 years, with only three out of eleven studies including patients over the age of 40 [7].

More research is needed to contribute to the understanding of how clinical factors such as patient age influence the treatment outcome of partial pulpotomy. The aim of this study was therefore to retrospectively assess clinical and radiographic outcomes of partial pulpotomies in permanent teeth with carious pulp exposure, which were performed without any age restriction.

## Material and methods

### Ethical approval and informed consent

The study was conducted in accordance with the Declaration of Helsinki (2013) and the regulatory requirements of the Swiss Human Research Act and Human Research Ordinance.

The study protocol was approved by the local ethics review board (EKNZ 2019–01,261). Patients gave their written informed general consent regarding the use of anonymized patient data for research purposes.

### Data retrieval

Data were collected from records of patients undergoing treatment between 2010 and 2019 at the undergraduate dental clinic of a university center for dental medicine. All patient records in which the procedures “direct pulp capping” (system code 4401/4.4010) or “vital tooth therapy” (system code 4402/4.4020) had been performed were identified using the accounting application tool of the electronic patient administration software. A total of 191 patient records were eligible for detailed hand search. They were screened by two investigators (FE, LKZ) according to the inclusion and exclusion criteria listed in Table 1. Clinical data of the initial treatment and of the latest available follow-up were extracted in a standardized manner using an encrypted spreadsheet. The following clinical parameters were recorded: probing depth, cold testing, pain on percussion, symptoms, discoloration, pulp capping material, cavity liner, and restoration material. Intraoral radiographs taken prior to partial pulpotomy, immediately after and at follow-up were assessed and compared using a monitor suitable for radiographic diagnostics. The parameters no change, hard tissue barrier formation, narrowing of pulp canal space, and apical lesion were recorded as applicable. Unclear cases were marked and discussed until an agreement was reached. Data encoding was implemented from the very outset of the data retrieval, with a senior dentist who was not otherwise involved in the project responsible for encoding. The dataset generated and analyzed during the current study is available from the corresponding author on request.

**Table 1:** Inclusion and exclusion criteria

Inclusion criteria	
1	General consent given to use anonymized treatment-related data
2	Partial pulpotomy received with at least 1-year follow-up
3	Reason for partial pulpotomy: carious pulp exposure
4	Clinical standard operating procedure followed
5	Radiograph available prior to and after partial pulpotomy and at follow-up
6	Clinical information on tooth sensitivity, signs and symptoms, and probing depth at baseline and follow-up
Exclusion criteria	
1	General consent not given or withdrawn to use anonymized treatment-related data
2	Partial pulpotomy performed as emergency treatment of irreversible pulpitis with subsequent root canal treatment or direct/indirect pulp capping with no pulp amputation
3	Observation less than 1 year*
4	Incomplete documentation at baseline or follow-up (no radiograph, partial data on the operative procedure and/or clinical parameters)

^*^Failures within the first year were recorded as failures and included in the analysis

### Treatment protocol partial pulpotomy

Upon entry in the undergraduate clinic, each patient received a complete oral examination including oral hygienic indices, radiographs, full dental status, tooth sensitivity testing, and complete periodontal probing depth assessment. An individual treatment plan was established. The preservation of tooth vitality was the main goal for teeth with deep carious lesions, provided there were no signs and symptoms of irreversible pulpitis present. At the end of the active treatment phase, patients were enrolled in regular follow-up appointments, with a recall interval between 3 and 12 months depending on the individual needs for supportive dental and periodontal care.

**Fig. 1:**
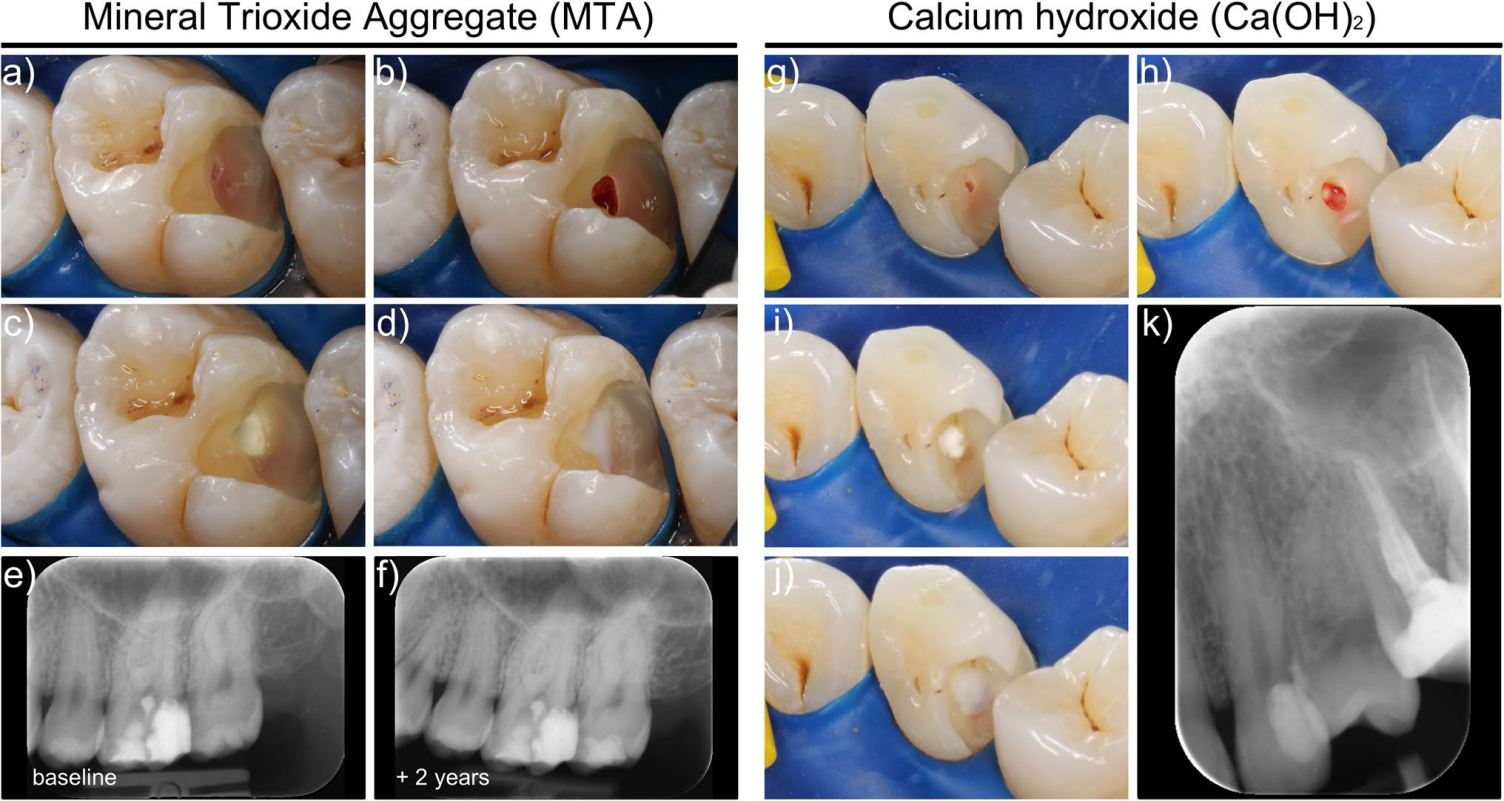
Representative photographs taken during partial pulpotomy procedure with either MTA (**a**–**f**) or Ca(OH)_2_ (**g**–**k**). **a**, **g** Exposed pulp after nonselective carious tissue removal to firm dentin; **b**, **h** pulpotomized area with bleeding control achieved; pulp capping with either **c** MTA or **i** Ca(OH)_2_ after partial pulpotomy; **d**, **j** light cured Ca(OH)_2_ cavity liner applied to cover the pulp capping material; **e**, **k** radiograph taken immediately after partial pulpotomy and the placement of a direct resin-based composite restoration; **f** periapical radiograph at follow-up after 2 years. No follow-up radiograph is available for the Ca(OH)_2_ case since this tooth ended in a failure after 22 days with signs of irreversible pulpitis and root canal treatment was performed

Partial pulpotomy in teeth with carious pulp exposure was carried out following an in-house standard operating procedure established in 2010. In brief, local anesthesia was administered and rubber dam isolation was mandatory at the beginning of caries removal. Nonselective carious tissue removal to firm dentin was performed by undergraduate dental students. Carious tissue removal in the pulpal aspects of the cavity was only undertaken when peripheral caries removal was completed. Teeth with pulp exposure during caries removal were disinfected with either 0.2% chlorhexidine digluconate (Dentohexin, Streuli Pharma AG, Uznach, Switzerland) or 1% sodium hypochlorite (NaOCl) and caries removal was completed (Fig. 1). Supervising resident or senior dentists performed the subsequent partial pulpotomy while students were assisting: a sterile diamond bur was used to remove the superficial part of the affected pulp tissue at the site of exposure (2–3-mm deep). Bleeding control was performed with a cotton pellet soaked in 1% NaOCl, which was pressed gently on the exposed pulp (Fig. 1). If hemostasis was attained within 1–5 min, the pulp was covered with either calcium hydroxide (Ca(OH)_2_) or mineral trioxide aggregate (MTA) (Fig. 1). MTA (ProRoot MTA, Dentsply Sirona, York, PA, USA or PD MTA White, Produits Dentaires, Vevey, Switzerland) was mostly used for premolars and molars. Ca(OH)_2_ (Sigma-Aldrich Chemie GmbH, Buchs, Switzerland) was used as capping material in some anterior teeth. The pulp capping material was placed using the MAP-System (Micro Apical Placement system, Produits Dentaires, Vevey, Switzerland) and condensed gently with sterile paper points. The capping material was covered with a light-curing resin-modified Ca(OH)_2_ cavity liner (Ultrablend Plus, Ultradent Products, South Jordan, UT, USA) (Fig. 1) in order to allow undergraduates to proceed with the conventional adhesive restorative protocol in the same visit. A three-step etch-and-rinse adhesive and resin-based composite were used to restore the tooth. A postoperative control radiograph was taken after the placement of the direct restoration (Fig. 1). If bleeding control was not achieved within 5 min, a successive layer of pulp tissue was removed, placing the surgical wound more cervically, and bleeding control was repeated for 5 min. In cases with persisting bleeding after the second partial pulpotomy, the pulp was diagnosed with irreversible pulpitis and root canal treatment was initiated.

### Statistical analysis

Data were sampled in Microsoft Excel and descriptive statistics were applied including counts and percentages for categorical variables and means and standard deviations (SD) for metric variables. Data regarding success and failure were processed and analyzed using the GraphPad Prism software package version 9.0.0 (GraphPad Software, San Diego, CA, USA). Three age groups were defined for age group differentiation analysis: < 30 years, 30–40 years, and > 40 years. Differences between the age groups were analyzed using the Mantel-Cox chi square test and Kaplan–Meier figures were presented for failures derived from survival analysis of censored data. The level of significance was set at α = 0.05.

## Results

**Fig. 2:**
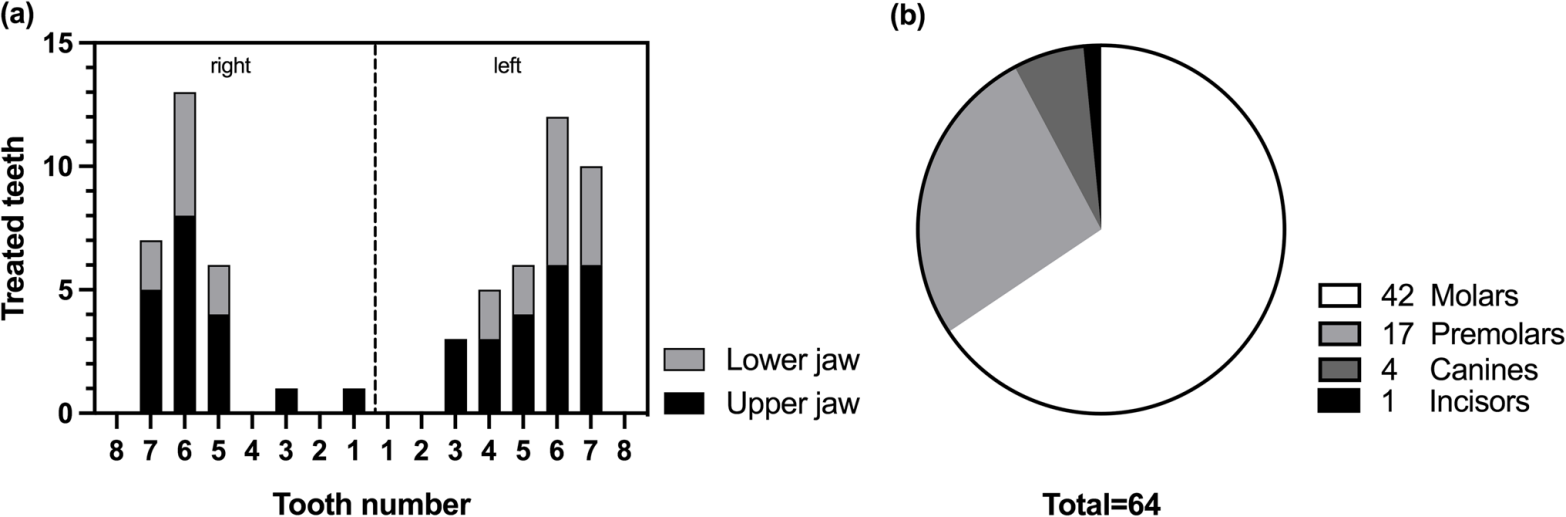
Overview of the tooth position and frequency of included partially pulpotomized teeth. **a** Visualization of all treated cases according to their tooth number of the upper and lower jaw divided into the patients’ right and left side. **b** Molars and premolars were the teeth most frequently treated, while only 4 canines and 1 central incisor were partially pulpotomized

In total, 154 patient records were assessed. Partial pulpotomy was performed in 111 cases. Sixty-four cases (58%) with a presumptive diagnosis of normal pulp or reversible pulpitis met the inclusion criteria (Table 2). Twenty cases (18%) were excluded owing to incomplete documentation (missing radiograph, partial data on the operative procedure and/or clinical parameters) and 27 cases (24%) were lost to follow-up. The tooth position of included partially pulpotomized teeth is presented in Fig. 2. Molars were the most frequently treated teeth (n = 42), followed by premolars (n = 17) and canines (n = 4), while only one central incisor was partially pulpotomized (Fig. 2). MTA was used predominantly as capping material (n = 63) with only one tooth, a canine, in the Ca(OH)_2_ group.

**Table 2:** Overview of screened records with percentage (%) of included and excluded cases according to the eligibility criteria

Procedure	2010–2016*	2017–2019^‡^	Total	%
Total screened records	109	45	154	
Full pulpotomy as emergency treatment prior to RCT^†^	29	14	43	–
Partial pulpotomy, complete documentation	55	9	64	58%
Partial pulpotomy, incomplete documentationº	9	11	20	18%
Partial pulpotomy, loss to follow-up	16	11	27	24%
Total number of partial pulpotomy	80	31	111	100%

^†^Treatment procedure registered with the same accounting position

ºMissing radiograph at baseline/follow-up, follow-up < 1 year

^*^Pos. 4401/4402

^‡^Pos 4.4010/4.4020

*RCT*, root canal treatment

**Fig. 3:**
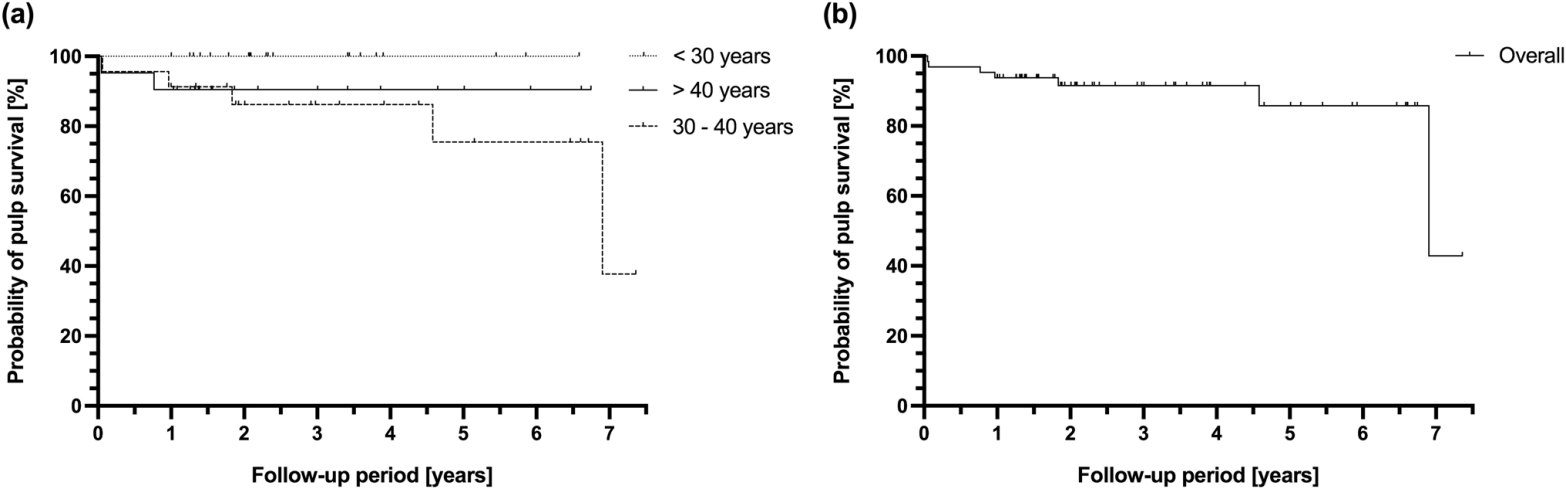
Kaplan–Meier survival curves after partial pulpotomy. **a** Probability of pulp survival in patients < 30 years of age, between 30 and 40 years, and > 40 years of age. Patients’ age at the time of the procedure did not affect the success rate of preserving pulp vitality after partial pulpotomy (p = 0.0225). **b** Overall probability of pulp survival

Table 3 summarizes patient-related factors and clinical parameters recorded prior to treatment and at the latest follow-up. The mean age at time of partial pulpotomy was 37.3 (± 13.5) years, with a median of 34 years (age range 18–85 years). The mean observation period was 3.1 (± 2.0) years, with a maximum observation period of 7.4 years. The overall success rate of preserving tooth vitality was 89.1%. A total of seven cases resulted in failure (10.9%). Four of seven failures occurred within the first year and were diagnosed with acute irreversible pulpitis (× 1), acute apical periodontitis (× 1), and chronic apical periodontitis (× 2) (Table 4). The remaining three failures occurred after 1.8 years (chronic apical periodontitis), 4.6 years (split tooth), and 6.9 years (acute irreversible pulpitis). The split tooth had to be extracted during the observation period resulting in a survival rate of 98.4% for partially pulpotomized teeth. No statistical significant difference was observed for the cumulative pulp survival rate between the age groups < 30 years (100%; *n* = 20), 30–40 years (75.5%, *n* = 23), and ≥ 40 years (90.5%, *n* = 21) for partial pulpotomy in teeth with carious pulp exposure (p = 0.225; Fig. 3). Subjects between 30 and 40 years of age showed a 5.6 times increased risk of failure compared with the younger age group (p = 0.089, 95%CI 0.77, 43.41) and 1.5 times increased risk of failure compared with the older age group (p = 0.606, 95%CI 0.30, 7.75). The overall cumulative pulp survival rate was 93.8% after 1 year, 91.5% after 2 years, and 85.8% after 6 years. It dropped to 48.9% after 7.4 years (Fig. 3).

**Table 3:** Summary of patient-related factors and clinical parameters pre-treatment and at follow-up with counts and percentages for categorical variables and means and standard deviations (SD) for metric variables

Parameter	Overall (%)	Success (%)	Failure (%)
Teeth	64 (100.0)	57 (89.1)	7 (10.9)
Female	28 (43.8)	25 (89.3)	3 (10.7)
Male	36 (56.3)	32 (88.9)	4 (11.1)
Age mean ± SD	37.3 ± 13.5	36.6 ± 12.5	43.4 ± 20.5
Observation (years) mean ± SD	3.1 ± 2.0	3.2 ± 1.9	2.2 ± 2.6
Clinical parameters pre-treatment			
Probing depths < 4 mm	59 (92.2)	52 (81.3)	7 (10.9)
Probing depths ≥ 5 mm	5 (7.8)	5 (7.8)	0 (0)
Cold testing positive	59 (91.2)	54 (84.4)	5 (7.8)
Cold testing hypersensitive	5 (7.8)	3 (4.7)	2 (3.1)
Cold testing negative	0 (0)	0 (0)	0 (0)
No pain on percussion	57 (89.1)	52 (81.3)	5 (7.8)
Pain on percussion	7 (10.9)	5 (7.8)	2 (3.1)
No symptoms	59 (92.2)	53 (82.8)	6 (9.4)
Symptoms (pain, discomfort)	5 (7.8)	4 (6.3)	1 (1.6)
Tooth discoloration	0 (0)	0 (0)	0 (0)
Clinical parameters post treatment			
Probing depths < 4 mm	55 (85.9)	48 (75.0)	7 (10.9)
Probing depths ≥ 5 mm	9 (14.1)	9 (14.1)	0 (0)
Cold testing positive	56 (87.5)	55 (85.9)	1 (1.6)
Cold testing hypersensitive	3 (4.7)	1 (1.6)	2 (3.1)
Cold testing negative	5 (7.8)	1 (1.6)	4 (6.3)
No pain on percussion	59 (92.2)	57 (89.1)	2 (3.1)
Pain on percussion	5 (7.8)	0 (0)	5 (7.8)
No symptoms	57 (89.1)	57 (89.1)	0 (0)
Symptoms (pain, swelling, discomfort, fistula)	7 (10.9)	0 (0)	7 (10.9)
Tooth discoloration	2 (3.1)	1 (1.6)	1 (1.6)
Treatment at follow-up			
None	57 (89.1)	57 (89.1)	0 (0)
Root canal treatment (RCT)	6 (9.4)	0 (0)	6 (9.4)
Extraction	1 (1.6)	0 (0)	1 (1.6)
Radiographic findings			
No changes	39 (60.9)	37 (57.8)	2 (3.1)
Hard tissue barrier formation	14 (21.9)	14 (21.9)	0 (0)
Narrowing of pulp canal space	7 (10.9)	6 (9.4)	1 (1.6)
Apical lesion	4 (6.3)	0 (0)	4 (6.3)
Tooth survival	63 (98.4)	57 (89.1)	6 (9.3)

**Table 4:** Details of the seven cases in which partial pulpotomy was unsuccessful

Diagnosis at follow-up	Tooth (FDI)	Time after PP	Sign/symptom prior to PP	Cold testing results prior to PP	Capping material	Age at PP (years)	Treatment at follow-up
Acute apical periodontitis	#27	17 days	Pain on percussion	Hypersensitive	MTA	57	RCT
Acute irreversible pulpitis	#23	22 days	None	Normal	Ca(OH)_2_	34	RCT
Chronic apical periodontitis	#35	280 days	None	Hypersensitive	MTA	85	RCT
Chronic apical periodontitis	#16	353 days	None	Normal	MTA	31	RCT
Chronic apical periodontitis	#15	1.8 years	None	Normal	MTA	34	RCT
Tooth fracture (split tooth)	#37	4.6 years	Pain on percussion	Normal	MTA	32	Extraction
Acute irreversible pulpitis	#26	6.9 years	None	Normal	MTA	31	RCT

*FDI*, Fédération Dentaire Internationale (World Dental Federation); *MTA*, mineral trioxide aggregate; *PP*, partial pulpotomy; *RCT*, root canal treatment

Radiographic findings revealed no changes within the pulp space in 39/64 cases (60.9%), a hard tissue barrier formation was observed in 14/64 cases (21.9%), and narrowing of the pulp canal space occurred in 7/64 cases (10.9%). Tooth discoloration was present in 2/64 cases (3.1%) (Table 3). Tooth discoloration was observed in a maxillary central incisor and a maxillary second premolar, in which MTA had been used as capping material.

## Discussion

This retrospective study, comprising data of 64 cases, aimed to assess clinical and radiographic outcomes of partial pulpotomy performed in permanent teeth with carious pulp exposure with a follow-up period of 1 year or more. The overall success rate of maintaining pulp vitality was 89.1% with 98.4% tooth survival. No significant difference was observed between the age groups < 30 years, between 30 and 40 years, and > 40 years even though the age of patients in whom partial pulpotomy was unsuccessful tended to be higher.

According to a recent meta-analysis analyzing data of five randomized controlled trials and six prospective clinical trials, the success rate of partial pulpotomies in permanent teeth with carious pulp has been estimated at 96% after 1 year and 92% after 2 years [7]. Outcome data of longer follow-up periods are scanty, but successful treatments of partial and full pulpotomies in asymptomatic and symptomatic (irreversible pulpitis) teeth were reported to range from 76 to 87% over an observation period of 5 years [11, 12]. The findings of the current investigation, indicating a successful preservation of pulp vitality in 94% after 1 year and 92% after 2 years, are in accordance with these results. The overall cumulative pulp survival rate dropped to 86% after a 6-year follow-up period.

The treatment protocol used in the present study considered unattainable bleeding control and negative pulp testing as relevant prognostic factors for an unfavorable outcome. If negative pulp testing with pulp necrosis was present or bleeding control could not be achieved within 5 min after a second deeper pulpotomy, root canal treatment was initiated. Both criteria, cold testing and bleeding control, remain essential for the assessment of pulpal health and play a crucial role in the case selection of vital pulp therapy procedures [8, 13]. In the current study, 11% of all analyzed teeth showed preoperative pain on percussion and 8% mild to moderate pain. Both factors were not associated with negative outcomes. Preoperative signs of irreversible pulpitis as prognostic factor have been discussed controversially with regard to the outcome of partial pulpotomy in permanent teeth with carious pulp exposure [7]. Most studies summarized in literature reviews report comparable success rates for irreversible inflamed and normal/reversible inflamed pulps and recommend no restriction of treating symptomatic teeth with partial pulpotomy procedures [8].

Currently available data suggest that patient age has no significant impact, favorable or otherwise, on the outcome of partial pulpotomy in permanent teeth with carious pulp exposure, which is consistent with the findings of the present study [8, 9]. The majority of studies reviewed on this topic, however, involved a study population below the age of 30 [7]. Kang and colleagues [14] investigated success rates of teeth with reversible pulpitis in subjects divided into two age groups: < 40 years and ≥ 40 years. Of 104 teeth, 4 failures (13%) were reported after 1 year in the younger age group and none in the older leading to success rates of 87% and 100%, respectively [14]. The comparison of both groups revealed no statistically significant difference [14]. However, the inhomogeneity of the age groups with 81 teeth (< 40 years) versus 23 teeth (≥ 40 years) and the relatively short observation period of 1 year might have affected the outcome. Another study with a 5-year follow-up period of partially pulpotomized teeth with irreversible pulpitis divided their study population into three groups: < 20 years, 21–29 years, and ≥ 30 years [11]. The success rates were 75%, 81%, and 76%, respectively, with no statistically significant differences between groups [11].

Since most studies investigated subjects up to 30 years of age or set 40 years as cut-off value for age group differentiation, the participants of the current investigation were divided into three age groups: below 30 years, between 30 and 40 years, and above 40 years. Hence, the data can be compared to the above-mentioned studies showing similar findings on preserving pulp vitality. Even though the cumulative survival rates for pulp vitality reported in the present study appear different among the age groups, no statistical significance was found. Though age could be excluded as influencing factor regarding the prognosis of preserving pulp vitality, the hazard ratio of losing pulp vitality was 5.6 times higher in subjects between 30 and 40 years than in subjects younger than 30 years. The explanation for the higher failure rate in the medium age group compared with the oldest remains unclear and might need to be assessed in a larger population. The drastic drop of the overall cumulative pulp survival rate from 85.8 to 42.9% between the follow-up year 6.5 and 7 can be explained by the failure after 6.9 years and the few remaining follow-up cases.

A classification of immediate/early failures occurring within the first 2 months and late/delayed failures has been proposed regarding the clinical and radiographic presentation [8, 15]. Early failures are associated with an acute inflammatory process caused by persistent bacteria exacerbating with severe pain shortly after the procedure, whereas late failures are attributed to pulp necrosis and periapical pathology with or without bacterial involvement [8, 15]. In the current investigation, four of seven failures occurred during the first year of observation. Two subjects presented with severe pain within the first 3 weeks after partial pulpotomy and were classified as early failures. The diagnoses comprised acute apical periodontitis (persistent pain, negative cold testing, pain on percussion) and acute irreversible pulpitis (persistent pain, hypersensitive cold testing, pain on percussion). The remaining two presented with negative cold testing at recall combined with periapical lesions and were diagnosed with chronic apical periodontitis after 9.5 months and 11.5 months. Those were classified as late failures. The other late failures occurred after 1.8 years (chronic apical periodontitis), 4.6 years (split tooth), and 6.9 years (acute irreversible pulpitis). The failure after 6.9 years with acute irreversible pulpitis raises the question of the underlying causes of the failure. A coronal leakage might be the reason for the bacterial invasion; however, the resin-based composite restoration was judged sufficient at time of failure.

Complete pulpotomy and partial pulpotomy obtain comparable success rates in teeth with a presumptive diagnosis of normal pulp or reversible pulpitis [7, 16]. However, clinical monitoring of pulpal health is more straightforward after partial pulpotomy because complete pulpotomy makes pulp sensibility testing challenging if not impossible. On the other hand, complete pulpotomy is regarded as technically less difficult and it may provide better restorative options in some cases compared with partial pulpotomy [8]. Given the paucity of studies comparing selective caries removal with partial or complete pulpotomy, there is a need for clinical trials to investigate and compare the efficacy of the most promising operative management strategies for teeth with deep carious lesions.

With few exceptions, Ca(OH)_2_ and MTA were used as capping material for anterior and posterior teeth, respectively, in the present study. The sample size and the small number of cases with visually detectable tooth discoloration precluded an assessment whether the capping material affected pulpal outcomes or the occurrence of discolorations. Moreover, one ought to consider that, in contrast to previous generations of MTA capping materials, the staining potential of tricalcium silicate materials which are available as capping agents today is virtually absent [8]. The standard operating procedure, followed in all cases included in the study, included the immediate provision of a direct adhesive restoration as a well-sealed restoration is crucial for pulpal health after capping.

Based on results from basic research and histologic studies, the presence of a mineralized dentin layer, a hard tissue barrier formation, beneath the damage is known to form following partial pulpotomy [10]. In a clinical setting, however, it might be difficult to detect such a hard tissue barrier formation owing to the degree of mineralization and over-projection of radiopaque restorative materials. Moreover, a hard tissue barrier formation is mainly a histologic entity that cannot be considered as a major success criterion.

Narrowing of the pulp canal space, indicative of hard tissue deposition by vital pulp tissue, may occur after partial pulpotomy [17]. It was radiographically observed in 7 cases (11%) in the present study, with one case requiring root canal treatment in the further course. The endodontic management of this tooth with a narrowed pulp canal space presented no significant challenge. However, to determine the rate and degree of pulp canal obliteration in teeth treated with partial pulpotomy, further studies with prolonged observation periods are needed.

The study had certain limitations that require careful consideration. First, this study was subject to the inherent limitations of any retrospective clinical investigation. Many undergraduates, residents, and senior dentists were involved in the delivery of dental care to patients included in the study. Standard operating procedures notwithstanding, operator-related factors were not as consistent as in prospective trials with calibrated operators. Moreover, the quality of the database relied on accurate recordkeeping and complete documentation. Eighteen percent of partial pulpotomy cases had to be excluded owing to an omitted radiographic assessment. For the present study, radiographic monitoring was crucial to comprehensively assess the treatment outcome. In clinical practice, however, the indication to take a radiograph is generally restricted in order to keep patients’ exposure to ionizing radiation as low as reasonably achievable. A radiograph was only taken when it was deemed expedient for diagnostic purposes, hence the number of excluded cases with missing radiographs. In addition, it is important to consider possible selection and attrition biases that may have occurred in the retrospective study. For instance, patients seeking dental treatment in an undergraduate clinic may, in some respects, not be representative of the general population. In the present study, 24% of patients were lost to follow-up and consequently data used to evaluate treatment outcomes were partial. The low recall rate of patients is a major limitation of this study.

Second, the restricted follow-up times are an important limitation of the study. The mean observation period in the present study was 3.1 years. Data from a systematic review suggest that 6 months or more may be considered as a suitable follow-up period to evaluate the success after a partial pulpotomy [7]. Nonetheless, studies with a more extended follow-up period are necessary to assess the long-term treatment outcome [8]. In particular, it is of interest to determine the rate of pulp obliterations that occur in the long run and to gain insight into the response of partially pulpotomized teeth to operative interventions undertaken in the restorative cycle at a later stage.

## Conclusion

An overall success rate of maintaining pulp vitality of 89.1% suggests that partial pulpotomy is a viable operative approach to treat permanent teeth with deep carious lesions. Patient age did not affect the treatment outcome in the present study.
